# Droplet-Assisted Microfluidic Fabrication and Characterization of Multifunctional Polysaccharide Microgels Formed by Multicomponent Reactions

**DOI:** 10.3390/polym10101055

**Published:** 2018-09-21

**Authors:** Nicolas Hauck, Nalin Seixas, Silvia P. Centeno, Raimund Schlüßler, Gheorghe Cojoc, Paul Müller, Jochen Guck, Dominik Wöll, Ludger A. Wessjohann, Julian Thiele

**Affiliations:** 1 Institute of Physical Chemistry and Polymer Physics, Leibniz-Institut für Polymerforschung Dresden e.V., D-01069 Dresden, Germany; hauck@ipfdd.de; 2 Department of Bioorganic Chemistry, Leibniz-Institut für Pflanzenbiochemie, D-06120 Halle (Saale), Germany; Nalin.deSeixasBorges@ipb-halle.de; 3 Institute of Physical Chemistry, RWTH Aachen University, D-52074 Aachen, Germany; centeno@pc.rwth-aachen.de (S.P.C.); woell@pc.rwth-aachen.de (D.W.); 4 Center for Molecular and Cellular Bioengineering, Biotechnology Center, Technische Universität Dresden, D-01307 Dresden, Germany; raimund.schluessler@tu-dresden.de (R.S.); gheorghe.cojoc@tu-dresden.de (G.C.); paul_mueller@tu-dresden.de (P.M.); jochen.guck@tu-dresden.de (J.G.)

**Keywords:** multicomponent reaction, polysaccharide microgels, droplet microfluidics, Passerini three-component reaction, Ugi four-component reaction

## Abstract

Polysaccharide-based microgels have broad applications in multi-parametric cell cultures, cell-free biotechnology, and drug delivery. Multicomponent reactions like the Passerini three-component and the Ugi four-component reaction are shown in here to be versatile platforms for fabricating these polysaccharide microgels by droplet microfluidics with a narrow size distribution. While conventional microgel formation requires pre-modification of hydrogel building blocks to introduce certain functionality, in multicomponent reactions one building block can be simply exchanged by another to introduce and extend functionality in a library-like fashion. Beyond synthesizing a range of polysaccharide-based microgels utilizing hyaluronic acid, alginate and chitosan, exemplary in-depth analysis of hyaluronic acid-based Ugi four-component gels is conducted by colloidal probe atomic force microscopy, confocal Brillouin microscopy, quantitative phase imaging, and fluorescence correlation spectroscopy to elucidate the capability of microfluidic multicomponent reactions for forming defined polysaccharide microgel networks. Particularly, the impact of crosslinker amount and length is studied. A higher network density leads to higher Young’s moduli accompanied by smaller pore sizes with lower diffusion coefficients of tracer molecules in the highly homogeneous network, and vice versa. Moreover, tailored building blocks allow for crosslinking the microgels and incorporating functional groups at the same time as demonstrated for biotin-functionalized, chitosan-based microgels formed by Ugi four-component reaction. To these microgels, streptavidin-labeled enzymes are easily conjugated as shown for horseradish peroxidase (HRP), which retains its activity inside the microgels.

## 1. Introduction

**Figure 1:**
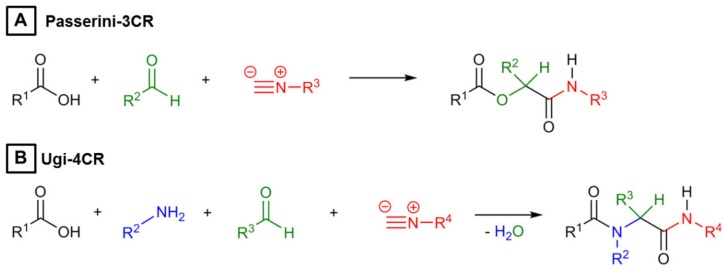
(**A**) Reaction scheme of a Passerini-3CR involving carboxylic acid (black), aldehyde (green), and isocyanide (red). (**B**) Reaction scheme of an Ugi-4CR with an additional amine component (blue).

Hydrogels are three-dimensional, crosslinked networks of hydrophilic polymers swollen in water. When processed into particles in the size range from 100 nm to 100 µm, these hydrogels are referred to as microgels according to IUPAC [1]. Compared to conventional bulk gels, microgels exhibit a very high surface-to-volume ratio resulting in a highly efficient mass transport to and from their polymer matrix [2]. As material basis, polysaccharides including hyaluronic acid, alginate, chitosan, or heparin are of special interest in microgel design, because they are non-toxic, biocompatible, non-immunogenic, often biodegradable, and allow for tuning their pore size and thus diffusivity over a large range [3]. These properties render polysaccharide-based microgels most suited for numerous applications in biotechnological and biomedical applications. These include the immobilization of DNA in hyaluronic acid microgels serving as an experimental platform with cell-mimicking properties [4,5], the trapping of biomolecules like enzymes in agarose-based microgels [6], the delivery of compounds via carboxymethylcellulose-based microgels [7], and cell culturing inside hyaluronic acid- and heparin-based microgels by attaching cell binding sites such as fibrinogen, the tripeptide sequence of arginine, glycine, and aspartate (RGD), as well as others [8,9]. While copper(I)-catalyzed azide-alkyne click chemistry [10,11], Diels-Alder cycloaddition [12,13], and the thiol-Michael addition reaction [14,15,16] have been employed for fabricating polysaccharide microgels in quantitative yield and under mild conditions, introducing multi-functionality in these materials usually requires elaborate chemical pre-modifications of the polysaccharide-based gel building blocks. These modifications are often time-consuming involving several synthesis and purification steps [17,18]. Moreover, owing to the limited number of different functional groups within the polysaccharide structure, one conjugation chemistry is frequently used for both crosslinking and introduction of functionality, which requires performing these reactions in a consecutive fashion to avoid competitive reactions. Therefore, it is desirable to enable crosslinking of the microgel network and incorporation of functional groups in a combined step in parallel. For that, multicomponent reactions like the Passerini three-component (P-3CR) and the Ugi four-component reaction (U-4CR) are well suited, as was recently demonstrated with the simultaneous (cyclo-)ligation and functionalization of peptides [19]. In case of the P-3CR, a carboxylic acid, an aldehyde, and an isocyanide component are condensed to yield α-(acyloxy) amides. The addition of a fourth component, a primary amine, leads to α-(acylamine) amides, as yielded by U-4CR [20]. Corresponding chemical structures starting from the individual components are depicted in Figure 1.

In bulk, the synthesis of non-functional hyaluronic acid-based [21,22], alginate-based [23,24], and chitosan-based [25] hydrogels has already been shown by means of P-3CR and U-4CR by Crescenzi et al. and Nyström et al. In contrast to ester-like crosslinking sites generated by P-3CR, amide-like bonds of U-4CR are known to be similarly stable at alkaline conditions [26]. However, just low molecular-weight crosslinkers, for instance glutaraldehyde [22,25], 1,5-diaminopentane [23,24,27], or l-lysine ethyl ester [21,27], were used, and the impact of higher-molecular weight crosslinker molecules was not studied. The synthesis of submicron-sized microgels by U-4CR based on carboxymethyl cellulose [28] and pectinic acid [29], which were crosslinked by low molecular-weight diisocyanides, was investigated by Mironov et al.

Here, we report the single-step synthesis of microns-sized polysaccharide-based multifunctional microgels by multicomponent reactions, specifically P-3CR and U-4CR. By choosing a polysaccharide as the base component, a homobifunctional poly(ethylene glycol) as crosslinker component, a heterobifunctional third component as functionalization agent, and an isocyanide as the reaction-starting component, the single-step synthesis of functional polysaccharide microgels is reported. Droplet-based microfluidics is employed for processing these materials into non-colloidal microgels with low size distribution down to 1 to 2% [30]. As this technique allows for efficient mixing of multiple components on the micron-scale, our polysaccharide-based microgels exhibit uniform porosity, elasticity and distribution of functional groups, and are thus well-suited for designing tailored microenvironments for (on-surface) cell cultures as well as experimental platforms for enzymatic cascade reactions and cell-free biotechnology.

## 2. Materials and Methods

### 2.1. Materials for Microgel Synthesis

All chemicals are used without further purification, unless stated otherwise. Alginate sodium salt, formaldehyde (37%), hydrochloric acid (HCl), dimethyl sulfoxide (DMSO), 3,3’,5,5’-tetramethylbenzidine (TMB), and hydrogen peroxide (30% *w*/*w*) were purchased from Sigma Aldrich/Merck, St. Louis, MO, United States. Hyaluronic acid sodium salt (40–65 kDa) was purchased from LifeCore, Kloten, Switzerland. Chitosan (90/30/A1) was purchased from BioLog Heppe, Landsberg, Germany. Poly(ethylene glycol) diamine with molecular weights of 2000, 1000, and 600 g mol^−1^, as well as poly(ethylene glycol) dialdehyde with a molecular weight of 1000 g mol^−1^, and carboxylic acid poly(ethylene glycol) biotin with a molecular weight of 1000 g mol^−1^ were purchased from Creative PEGWorks, Chapel Hill, NC, United States. *Tert*-butyl isocyanide was purchased from TCI Deutschland, Eschborn, Germany. Streptavidin Alexa Fluor 555 conjugate was purchased from Invitrogen, Carlsbad, CA United States. Streptavidin-HRP DY998 was purchased from R&D Systems, Minneapolis, MN, United States. Deionized water from a Milli-Q water purifier with a resistance of 18.2 MΩ cm was used.

### 2.2. Microfluidic Device Fabrication and General Microfluidic Experimental Setup

Microfluidic flow cells are manufactured by employing combined photo and soft lithography. A master template is first created by spin-coating a negative photoresist (SU-8 25, Mirochem. Co., Westborough, MA, USA) onto the polished side of a 3 inch silicon wafer (Siegert Wafer, Aachen, Germany). A mask aligner (MJB3, Süss MicroTec, Garching, Germany) is used to illuminate the resist with the desired microchannel structure by the help of a printed photomask. The non-illuminated parts of the photoresist are removed with a developer (mr-Dev 600, Micro Resist Technology, Berlin, Germany). To check for a uniform height of 50 µm of the microchannel structure, the master templates are characterized by a confocal microscope (µsurf expert, NanoFocus AG, Oberhausen, Germany). Channel width and height at the droplet-forming, flow-focusing nozzle are 50 µm. Subsequently, a polydimethylsiloxane (PDMS) base and crosslinker mixture (Sylgard 184 silicone elastomer kit, Dow Corning, Midland, TX, USA) is combined at a ratio 10:1 (*w*/*w*), mixed and degassed by a planetary centrifugal mixer (ARE-250, Thinky, Laguna Hills, CA, USA), poured onto the microfluidic master device surface, and polymerized in an oven at 65 °C for 1 h. Then, the PDMS replica is peeled off the master, the microstructures cut out of the PDMS layer with a scalpel, and access ports for tubing are punched into the soft replica with a biopsy punch (diameter: 1.0 mm, KAI Medical, Solingen, Germany). Finally, the PDMS block containing the microchannels is attached to a glass slide (76 × 26 mm) after oxygen plasma treatment (100 W, 20 s, MiniFlecto 10, Plasma Technology GmbH, Herrenberg, Germany). To provide a hydrophobic microchannel surface, a solution of (tridecafluoro-1,1,2,2-tetrahydrooctyl)trichlorosilane (1 *v*/*v* %) (Gelest) in a fluorinated oil Novec 7500 (IoLiTec GmbH, Heilbronn, Germany) is injected into the channels and left for solvent evaporation in air until device usage.

For all microfluidics experiments, microfluidic devices are connected to high-precision syringe pumps (Harvard Apparatus Pump 11 Pico Plus Elite, Harvard Apparatus, Holliston, MA, USA) via PE tubing (inner diameter: 0.38 mm, outer diameter: 1.09 mm, Hartenstein GmbH, Würzburg, Germany). 500 µL gastight syringes (1750 TLL SYR, Hamilton, Franklin, IN, USA) and 3 mL disposable syringes (BD Luer-Lok tip, Becton Dickinson, Franklin Lakes, NJ, USA) are used for the dispersed and continuous phases, respectively. To monitor droplet formation, microflow cells are operated on an inverted bright-field microscope (Primovert, Carl Zeiss AG, Oberkochen, Germany), equipped with a 10× objective lense (air), and coupled to a high-speed digital camera (Miro eX4, Vision Research Inc., Wayne, MS, USA). Bright-field and fluorescence microscopy imaging of droplets and microgels is performed on an inverted microscope (DMi8, Leica, Wetzlar, Germany).

### 2.3. Microfluidic Fabrication of P-3CR and U-4CR Microgels

#### 2.3.1. General Procedure of Microgel Fabrication

For the fabrication of P-3CR microgels, the carboxylic acid and aldehyde components are dissolved in deionized water and acidified with 2 M hydrochloric acid (HCl) to reach a pH of 3.5 as microgel precursor solution, also referred to as the dispersed phase (DP) in the microfluidic experiment. For the fabrication of U-4CR microgels, an additional amine component is added. All reaction mixtures serving as DPs, are homogenized for 30 min in a thermoshaker (800 rpm, 22 °C). The two continuous phases (CP) of the microfluidic experiment are prepared equally for every experiment as follows. CP1, which is emulsifying the aqueous gel precursor droplets at the first junction, consists of a home-made triblock copolymer surfactant [31] (Krytox-Jeffamine-Krytox, 2.5% *w*/*w*) dissolved in the fluorinated oil Novec 7500. CP2, added at the second microfluidic cross-junction, contains *tert*-butyl isocyanide (12% *v*/*v*) in addition to the surfactant solution in Novec 7500, which diffuses into the aqueous droplets formed at the first cross-junction to initiate microdroplet-to-microgel conversion. The resulting emulsions are collected in fractions at intervals of 10 min in Eppendorf tubes, which are sealed with parafilm to prevent evaporation, and kept for 48 h at room temperature to ensure complete crosslinking of the microgels. Afterwards, the fluorinated oil phase containing the surfactant and isocyanide is removed with a syringe, 250 µL of deionized water is added, and the microgels are transferred into water by washing with 250 µL of Novec 7500 containing 20% *v*/*v* of 1H,1H,2H,2H-perfluorooctanol (PFO, Sigma Aldrich/Merck, St. Louis, MO, USA). The washing step is repeated two more times. To evaluate the initial microdroplet sizes and corresponding microgels, 150 droplets/microgels are measured manually using ImageJ, and mean diameters as well as standard deviations are calculated.

#### 2.3.2. Hyaluronic Acid-Based P-3CR Microgels

For the fabrication of Passerini microgels, 12 mg of low-molecular weight hyaluronic acid (sodium salt, 40–65 kDa) are dissolved in 200 µL deionized water. After complete dissolution, the solution is acidified to a pH of 3.5 by adding 5.3 µL of 2 M HCl. Subsequently, 12 mg of poly(ethylene glycol) dialdehyde (PEG-dialdehyde) (Mass average molar mass (M_W_): 1000 g mol^−1^) are added. Flow rates are 40 µL h^−1^ and 400 µL h^−1^ for the DP and each CP, respectively.

#### 2.3.3. Hyaluronic Acid-Based U-4CR Microgels

For all hyaluronic acid-based microgels, 12 mg of low-molecular weight hyaluronic acid (sodium salt, 40–65 kDa) are dissolved in 200 µL deionized water and acidified with 5.3 µL of 2 M HCl. Based on this, a 3-by-3 matrix of different microgel compositions is built up by adding 8 mg, 12 mg, or 16 mg of poly(ethylene glycol) diamine (PEG-diamine) as crosslinker of three different molecular weights (2000, 1000, and 600 g mol^−1^) to a threefold molar amount of formaldehyde (37% *v*/*v* in water, related to the amount of the respective PEG crosslinker), as depicted in Table 1, Table 2 and Table 3.

Flow rates for all hyaluronic acid-based U-4CR microfluidic experiments are 40 and 400 µL h^−1^ for the DP and each CP, respectively.

#### 2.3.4. Alginate-Based U-4CR Microgels

Prior to dissolving the polysaccharide, 198 µL of deionized water are acidified with 1.8 µL of 2 M HCl. This is crucial to prevent the formation of alginate acid gels by adding the HCl after dissolving sodium alginate. 8 mg of sodium alginate are then added to the 200 µL of acidified, deionized water. To improve dissolution, the precursor solution is sonicated for 20 min at 40 °C. 5.3 mg of PEG-diamine (M_W_: 1000 g mol^−1^) and then 1.2 µL of formaldehyde (37% *v*/*v* in water) are added. Flow rates are 30 and 500 µL h^−1^ for the DP and each CP, respectively.

#### 2.3.5. Chitosan-Based U-4CR Microgels

For preparing chitosan-based microgels, 184 µL of deionized water are added to 6 mg of chitosan to form a suspension. By adding 15 µL of 2 M HCl and 0.7 µL of acetic acid, serving as the carboxylic acid compound of the Ugi reaction, the chitosan is finally dissolved by sonication for 20 min at 40 °C. Thereafter, 4 mg of PEG-dialdehyde (M_W_: 1000 g mol^−1^) are added. Due to the high viscosity of the DP, flow rates are 20 and 600 µL h^−1^ for the DP and the CPs, respectively.

#### 2.3.6. Chitosan-Based, Biotin-Functionalized U-4CR Microgels

6 mg of chitosan are suspended in 185 µL of deionized water. Thereafter, 15 µL of 2 M HCl are added and chitosan dissolved by sonication for 20 min at 40 °C. After complete dissolution of chitosan, 4 mg of PEG-dialdehyde (M_W_: 1000 g mol^−1^) are added and dissolved in the polysaccharide solution in a thermoshaker (1000 rpm, 25 °C). To complete the precursor solution preparation, 12 mg of carboxylic acid poly(ethylene glycol) biotin (M_W_: 1000 g mol^−1^) are added and mixed for approximately 10 min in a thermoshaker (1000 rpm, 60 °C) until complete dissolution. Flow rates are 40 and 400 µL h^−1^ for the DP and each CP, respectively.

### 2.4. Immobilization and Activity Assay of Horseradish Peroxidase (HRP)

50 µL of chitosan-based, biotin-functionalized U-4CR microgel suspension (approximately 500,000 microgels per mL) in deionized water are incubated overnight in a thermoshaker (4 °C, 200 rpm) with 0.05 µM of horseradish peroxidase (HRP) streptavidin conjugate. To remove non-immobilized HRP, microgels are washed two times with 400 µL deionized water, concentrated by centrifugation (1 min, 4200 rcf), and supernatant is removed.

The activity of HRP is measured by the oxidation of 3,3′,5,5′-tetramethylbenzidine (TMB) [32]. The assay is performed in a 96-well plate TECAN Infinite 200 PRO microplate reader. Each measurement is performed in triplicate, and the absorbance at 653 nm is recorded for a period of 15 min. For a typical approach with a final volume of 100 µL, 5 µL of 15 mM H_2_O_2_ and 5 µL of 2 mM TMB in dimethyl sulfoxide (DMSO) are added to 80 µL of deionized water. Depending on the experiment, 10 µL of deionized water for the blank, 10 µL of 0.05 µM HRP conjugate, 10 µL of previously prepared HRP-functionalized microgels, or 10 µL of the supernatant are added, the solutions or microgel suspensions mixed, and the measurements are immediately started. The blank values are later subtracted from all measurements.

### 2.5. Colloidal Probe Atomic Force Microscopy (CP-AFM)

All colloidal probe atomic force microscopy (CP-AFM) indentation experiments are performed on a MFP-3D Bio (Asylum Reasearch, Oxford Instruments, Abingdon-on-Thames, UK) mounted on an inverted optical microscope (Axio Observer Z1, Zeiss, Oberkochen, Germany). For elasticity measurements, the cantilever (CSC38, no Al, tipless, nominal spring constant = 0.09 N m^−1^, Mikromasch, Sofia, Bulgaria) is calibrated by determining the inverse optical lever sensitivity (InvOLS) by line fitting of the repulsive part of force-distance (F-D) curves on a glass microscope slide. Assuming non-deformability of the glass surface, a direct correlation between cantilever deflection in nm-units and displayed output voltage is established. The spring constant of each cantilever is calibrated by the thermal noise method [33] before a 20 µm-diameter, native silica sphere is glued onto the free-standing end of cantilever with a two-component epoxy glue (UHU endfest, UHU, Bühl, Germany) using a micromanipulator (MP-285, Sutter Instruments, Novato, CA, USA) and a microscope (MZ9.5, Leica, Wetzlar, Germany).

Before sample preparation, the glass substrate of the fluid cell is cleaned with isopropanol and water and treated together with the colloidal probe cantilever in an oxygen plasma for 1 min (80 W). To enhance the adhesion of the negatively charged hyaluronic acid-based U-4CR microgels, the glass substrate is coated with a poly(ethylene imine) layer. Afterwards, 10 µL of the microgel suspension are spread on the modified glass substrate, and the fluid cell is immediately filled with 4 mL of deionized water to prevent drying of the microgels before and during the AFM measurements. To locate the microgels in the fluid cell, an inverted optical microscope and the top-view optics of the AFM are used. The colloidal probe is pre-aligned centrically above each microgel by optical control with the microscope, and precise axissymmetric contact between the probe and sample is ensured by determining the gel apex by recording spatially resolved force-distance (F-D) curves. The force-set point for these F-D curves is adjusted individually for each microgel type to achieve sufficient deformation for mechanical evaluation (≥500 nm). The cantilever speed is 2 µm s^−1^. For every microgel type, five F-D curves per microgel are recorded for 20 individual microgels. Possible InvOLS variations are controlled after the measurement of 5 microgels.

All raw data measured as movement of the z-piezo and the deflection signal of the cantilever on a segmented photodiode, is processed and evaluated in a home-made IgorPro procedure according to [34]. The Johnson-Kendall-Roberts model [35] is used to fit the F-D curves to account for the presence of adhesive interactions between probe and microgel.

### 2.6. Confocal Brillouin Microscopy

The Brillouin shift is measured by confocal Brillouin microscopy, using a custom built set-up, as described previously [36]. The basic principle of the technique has been described in [37,38]. Equipped with a 40×/1.1NA objective, spatial resolution of less than 1 µm in lateral plane and approximately 3 µm in axial direction is achieved. To acquire spatially resolved images, an automatic translation stage is employed. For the measurement, microgel samples are prepared in cavities created by an imaging spacer (diameter: 9 mm, thickness 0.12 mm, Grace Bio-Labs SecureSeal, Sigma Aldrich, St. Louis, USA) between two glass slides. To prevent the microgels from being dragged by the laser beam, glass slides are pre-modified with poly(ethylene imine) to enhance their adhesion to the surface. The Brillouin shift is then measured along a line through the center of the microgels with a step size of 0.5 µm. In case of Brillouin shift maps of single microgels, an area of 120-by-120 µm is mapped with a step size of the translation stage of 1.5 µm.

### 2.7. Refractive Index Measurement

Quantitative phase images are recorded in a similar way as described in Schürmann et al. [39], a setup based on a standard inverted microscope (IX71, Olympus, Shinjuku, Japan) is used. A halogen lamp (TH4-200, Olympus), having the spectrum restricted by a band pass filter to 647 ± 57 nm (F1, F37-647, AHF Analysentechnik, Tübingen, Germany), is used as a light source for quantitative phase image acquisition. A 40×-microscope objective is used for imaging (NA 0.65, Zeiss). A commercial quantitative phase imaging camera (SID4-Bio, PHASCIS, St. Aubin, France) is mounted on the side port of the microscope. A telescope (L1, LD2060-A f = −15 mm and L2, LBF254-075-A f = 75 mm, Thorlabs, Newton, MA, USA) is implemented for a magnified image on the camera.

Quantitative phase images are processed using a custom built software, DryMass version 0.1.4 (https://drymass.readthedocs.io/en/0.1.4/index.html), as described in [39]. Refractive index distributions of microgel beads are obtained by inferring spherical symmetry as described in [40] using DryMass. Cross-sectional refractive index profiles are obtained from quantitative phase images by, again, inferring spherical symmetry and employing optical diffraction tomography as implemented in the Python package ODTbrain version 0.2.1 [41]. From the resulting three-dimensional refractive index map of one microgel bead, all center sections within a 5 µm wide interval relative to the reconstruction center are extracted and averaged. To reduce noise, the averaged section is filtered with a 3 × 3 px median filter and subsequently averaged radially. The radial refractive index profile corresponds to the values in the reconstructed slice along the coordinate axes.

### 2.8. Fluorescence Correlation Spectroscopy (FCS)

Fluorescence correlation spectroscopy measurements [42,43] are conducted on microgels dispersed in deionized water. A Microtime setup from Picoquant (Berlin, Germany) with Symphotime software was used. For that, individual microgel dispersions are diluted with an aqueous solution of the fluorophore tetramethylrhodamine-dextran with a molecular weight of 70 kDa (Invitrogen, ThermoFisher Scientific, Waltham, MA, USA). The fluorophore is dissolved directly in deionized water and further diluted until reaching the optimal concentration for FCS measurements. The as-prepared microgel suspensions are used without additional filtration. FCS measurements are carried out at 25 µW of a pulsed 532 nm laser source at 22 °C, and the fluorescence is collected with an Olympus (Tokyo, Japan) UPLanApo 60× water-immersion objective with 1.20 NA and transmitted through a long-pass dichroic mirror (F67) and a band-pass filter (593/40) (both filters purchased from AHFanalysentechnik, Tübingen, Germany). Confocal detection is achieved with a 75 µm pinhole. After passage through the pinhole, the fluorescence emission is split into two paths by a 50/50 beam splitter (provided by Picoquant, Berlin, Germany) and detected by two single photon avalanche diode (SPAD) Excelitas detectors (provided by Picoquant, Berlin, Germany). Calibration of the confocal volume is realized by curve fitting of the FCS curve of a Rhodamine 6G solution in water (D(Rh6G) = 4.14 × 10^−6^ cm^2^ s^−1^ [44]) and characterized by an effective volume of 1.049 fL.

All FCS measurements correspond to fluorescence time traces of 180 s. At least 18 different measurements are carried out for each microgel type. More specifically, FCS is first measured in an area outside of the microgels for the water dispersion to estimate the diffusion coefficients of the fluorescent species outside of the microgel. Subsequently, different microgels are selected, and FCS is measured approximately in the center of each particle and at 25 µm from the center at a height of approximately 40 µm over the glass cover slip surface. Since the delivered tetramethylrhodamine-labelled dextranes contain a significant amount of free dye (not bound to dextrane) in every experiment, the FCS curve are fitted with two diffusion components with the contributions *ρ*_1_ and *ρ*_2_, one for the labelled dextrane and one for the free dye, and triplet excitation [45] is taken into account by including a variable triplet contribution *T* with a fixed triplet lifetime *τ_T_* of 3 µs:

(1) $$G\left(t\right)=\left(1+T\left(exp^{-\frac{t}{\tau_{T}}}-1\right)\right)\cdot \left(\frac{\rho_{1}}{\left(1+\frac{t}{\tau_{D1}}\right)\sqrt{1+\frac{t}{\tau_{D1}\cdot \kappa^{2}}}}+\frac{\rho_{2}}{\left(1+\frac{t}{\tau_{D2}}\right)\sqrt{1+\frac{t}{\tau_{D2}\cdot \kappa^{2}}}}\right)$$

The value *κ* is a geometrical factor accounting for the non-spherical shape of the confocal volume. A *κ* value of 4.59, as determined from a calibration measurement, is used for all microgel samples. To reduce the number of free parameters during fitting, the diffusion coefficient of free fluorophore measured in the area outside of the microgels is considered to remain unchanged for the measurements inside the microgels, and accordingly, the diffusion time of this species is kept constant in the two-component FCS fitting.

## 3. Results and Discussion

### 3.1. Microgel Preparation by Multicomponent Reactions via Droplet Microfluidics

**Figure 2:**
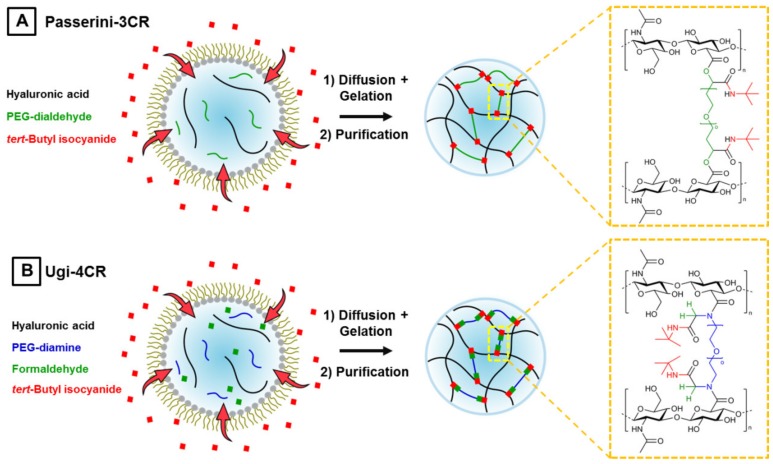
Schemes of droplet microfluidics-assisted production of hyaluronic acid-based microgels by means of Passerini three-component reaction and Ugi four-component reaction. (**A**) A water-in-oil emulsion of an aqueous solution of hyaluronic acid as microgel backbone and PEG-dialdehyde crosslinker is formed within a continuous phase made of fluorinated oil, *tert*-butyl isocyanide, and a home-made triblock copolymer surfactant to prevent coalescence of the as-formed droplets. The isocyanide component diffuses into the aqueous droplets through the surfactant layer to initiate the Passerini-3CR. After gelation, the microgels are purified by transferring them into an aqueous medium assisted by PFO that induces emulsion coalescence. The chemical structure of the crosslinking site is depicted within the yellow box. (**B**) Production of microgels by Ugi-4CR analogous to (**A**), but with formaldehyde instead of PEG-dialdehyde and a homobifunctional amine component, PEG-diamine, serving as crosslinker.

Multicomponent reactions like the Passerini three-component (P-3CR) reactions and especially the Ugi four-component reaction (U-4CR) are well-suited for the synthesis of polysaccharide hydrogels, since they are atom-efficient and economic crosslinking techniques, which do not require the pre-functionalization of microgel precursors. In case of hyaluronic acid-based P-3CR gels, hyaluronic acid reacts with PEG-dialdehyde and *tert*-butyl isocyanide to yield α-(acyloxy) amides at its crosslinking sites. The chemical structure of this crosslinking site in Figure 2A reveals that this hydrogel type is degradable at a pH higher than 7, since an ester-like bond forms, which is labile at basic conditions. This degradability opens possibilities for applications where a dissolution of the hydrogel network is of interest, but it also limits the overall usability of this microgel type. To overcome this issue, microgels formed by U-4CR can be employed, because they form from the same three components of the P-3CR plus an additional amine species, α-(acylamine) amides, which are not pH sensitive. For hyaluronic acid-based U-4CR microgels, homobifunctional PEG-diamine is used as crosslinker to ensure that the crosslinking sites are exclusively created by pH-stable Ugi bonds (Figure 2B), since the crosslinking can occur only via the bifunctional amine component. This is crucial, since the P-3CR also can occur in presence of amines and is consequently competitive to the U-4CR if formation of the imine intermediate is incomplete or reversed.

**Figure A1:**
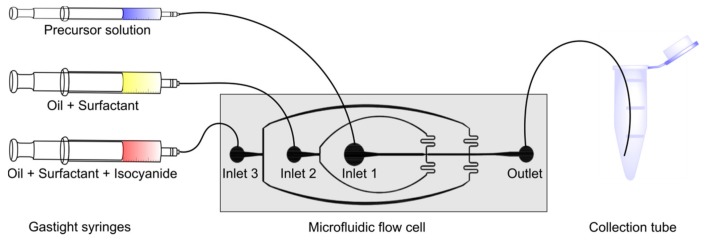
Scheme of experimental setup for droplet microfluidic fabrication of P-3CR and U-4CR microgels including gastight syringes, a microfluidic flow cell for droplet production, and a collection tube for collecting the formed water-in-oil emulsion.

With a focus on future application of our polysaccharide hydrogels in on-gel cell culturing and cell-free biotechnology, P-3CR and U-4CR are coupled with droplet-assisted microfluidic templating to generate tailored microgels. The corresponding flow cell is designed in AutoCAD to allow for separate injection of a dispersed phase, an aqueous mixture of dissolved hydrogel precursors, and two additional continuous phases, consisting primarily of a fluorinated oil, which is immiscible with the aqueous hydrogel precursor phase. A scheme of the experimental setup for droplet microfluidic fabrication of P-3CR and U-4CR microgels can be found in the Appendix Figure A1. At the first junction of the flow cell, droplets are pinched off the aqueous phase and stabilized in the continuous oil phase against coalescence by a home-made triblock copolymer surfactant (Krytox-Jeffamine-Krytox). Droplets are produced by geometrically controlled breakup from a continuous liquid stream exactly at the microfluidic cross-junction. This flow regime is characterized by both the capillary number of the continuous phase and the Weber number of the dispersed phase being <1 [46]. Direct addition of the isocyanide component for initiating the multicomponent reaction at the first junction could lead to spontaneous gelation directly at the first junction and accidental blockage of the microfluidic channel. Thus, the isocyanide component is added at a subsequent second junction. A solution of isocyanide in fluorinated oil, with the same surfactant concentration as at the first junction is fed into the second cross-junction. The chosen surfactant concentration of 2.5 wt %, which is above the critical micelle concentration of the home-made ABA surfactant of 1.8 wt % (according to T. Heida et al., unpublished data), leads to the formation of surfactant micelles in the continuous oil phase and thus to an increased micelle-driven exchange between droplets. The increase in permeability of the surfactant layer is beneficial for the droplet-encompassed multicomponent reactions, since diffusion of the isocyanide (e.g., inside micelles) into the precursor droplet is increased. For all experiments *tert*-butyl isocyanide is used as isocyanide component, because this compound shows high reactivity and also the highest water-solubility among a small set of tested commercial isocyanides (*tert*-butyl isocyanide, methyl isocyanoacetate, ethyl isocyanoacetate, and cyclohexyl isocyanide). All of the isocyanides exhibit high solubility in fluorinated oil, but only poor to none in water. By diffusion of *tert*-butyl isocyanide into the precursor droplets, the crosslinking reaction is initiated, and gelation is completed for all types of microgels within 48 h. The composition of the two continuous phases is identical for the P-3CR and U-4CR experiments. The aqueous phase differs slightly, consisting of a carboxylic acid component and an aldehyde component in case of the P-3CR, and an additional amine component in case of the U-4CR, respectively. For comparing P-3CR and U-4CR, 12 mg of the base material hyaluronic acid is dissolved for both types of reactions in deionized water, acidified with 2 M HCl. For the P-3CR, 12 mg of poly(ethylene glycol) (PEG)-dialdehyde (1000 g mol^−1^), for U-4CR, 12 mg of PEG-diamine (1000 g mol^−1^) are added to a threefold molar amount of formaldehyde (related to the amount of the respective PEG crosslinker) to form the aqueous dispersed phase for microfluidics experiments.

**Figure 3:**
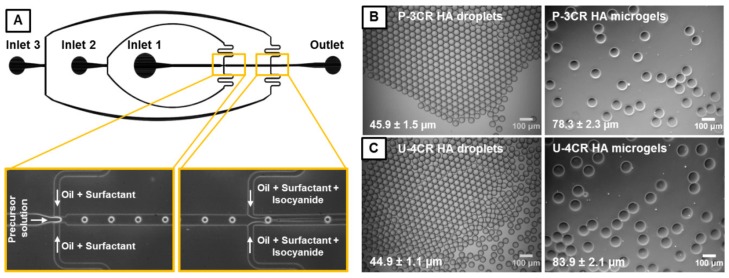
(**A**) AutoCAD-made design of microfluidic flow cell and corresponding phase-contrast microscopy images of the first junction, where droplets form from the precursor solution, and of the second junction, where the isocyanide component is added to initiate the multicomponent reactions. (**B**) Phase-contrast microscopy images of microemulsion droplets and corresponding microgels made by P-3CR, and (**C**) by U-4CR; both panels include mean and standard deviations.

**Figure A2:**
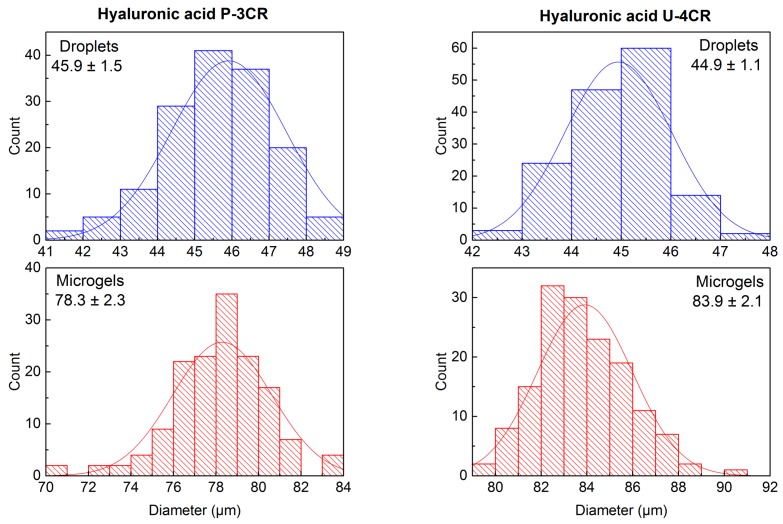
Size distribution histograms with additional mean and standard deviation for droplets and microgels of hyaluronic acid-based P-3CR microgels (**left**) and hyaluronic acid-based U-4CR microgels (**right**).

Bright-field microscopy images of precursor microdroplets (Figure 3B,C) indicate formation of microemulsions with a low dispersity, yielding droplet diameters of 45.9 ± 1.5 µm and 44.9 ± 1.1 µm for hyaluronic acid (HA)-based P-3CR and U-4CR microgels, respectively. The microgels maintain their low dispersity with values of 78.3 ± 2.3 µm and 83.9 ± 2.1 µm, respectively, after gelation and transfer into water. However, a 1.7-fold increase for P-3CR and a 1.9-fold increase for U-4CR microgels in diameter is observed, corresponding to a 6.8-fold increase for P-3CR and a 7.5-fold increase for U-4CR microgels in volume compared to their droplet template. These findings on the swelling behavior of hyaluronic acid-based gels are in accordance with the results of Crescenzi et al., who reported a 3-fold swelling for hyaluronic acid-based P-3CR, and a 9- to 28-fold swelling of hyaluronic acid-based U-4CR bulk gels, determined by comparing the weight of the freshly synthesized macrogel with its equilibrium weight in dialyzed water [25,27]. Size distribution histograms of hyaluronic acid-based P-3CR and U-4CR droplets and microgels are presented in Appendix Figure A2.

**Figure 4:**
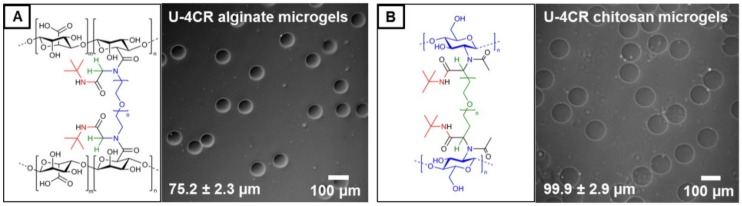
(**A**) Chemical structure of crosslinking site of alginate-based U-4CR microgels crosslinked by PEG-diamine and corresponding phase-contrast microscopy image, including mean and standard deviation of the microgels. (**B**) Chemical structure of crosslinking site of chitosan-based U-4CR microgels crosslinked by PEG-dialdehyde and corresponding phase-contrast microscopy image, including mean and standard deviation of the microgels.

**Figure A3:**
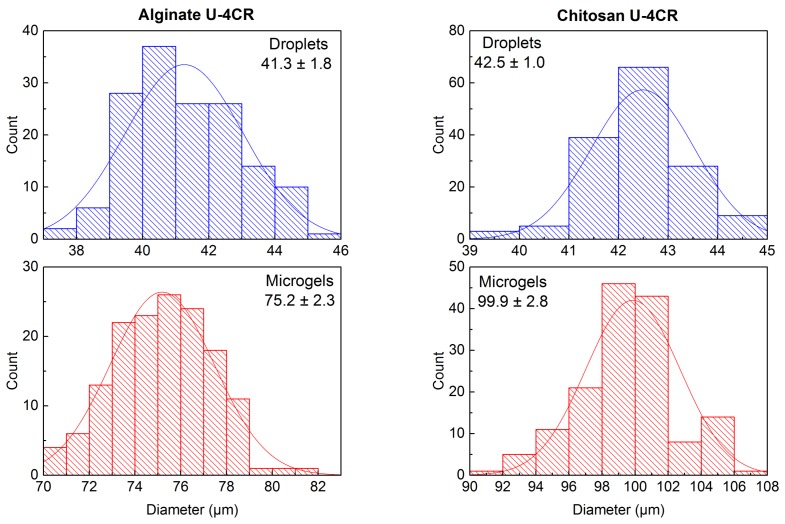
Size distribution histograms with additional mean and standard deviation for droplets and microgels of alginate-based U-4CR microgels (**left**) and chitosan-based U-4CR microgels (**right**).

Besides hyaluronic acid, U-4CR is likewise suited to form microgels from other polysaccharides by droplet microfluidics, including alginate and chitosan. In case of alginate, which also bears carboxylic acid groups just like hyaluronic acid, crosslinking is similarly induced by a homobifunctional PEG-diamine. Here, formaldehyde and *tert*-butyl isocyanide are equally used. The chemical structure of the corresponding alginate U-4CR crosslinking site is depicted in Figure 4A, together with a phase-contrast image of alginate microgels prepared by U-4CR, exhibiting the typical low dispersity of gel diameter in water with 75.2 ± 2.3 µm. With droplet sizes of 41.3 ± 1.8 µm, alginate-based U-4CR microgels reveal a 1.8-fold increase in diameter and a 7.3-fold increase in volume, which is comparable to hyaluronic acid-based microgels. For chitosan-based microgels, different components for crosslinking have to be chosen, since chitosan bears amines instead of carboxylic acids as intrinsic functional groups. PEG-dialdehyde is consequently selected to ensure crosslinking exclusively by Ugi bonds. Additional components for chitosan-based U-4CR microgel preparation are acetic acid as a low-molecular weight reaction component with fast diffusion characteristics similar to formaldehyde used for hyaluronic acid-based microgel preparation before, which is easily available at the crosslinking site, and the commonly used *tert*-butyl isocyanide. Figure 4B shows the chemical structure of the crosslinking site and a typical phase-contrast microscopy image of the microgels after transfer into water, revealing a low dispersity with diameters of 99.9 ± 2.9 µm. With droplet sizes of 42.5 ± 1.0 µm, there is thus a 2.4-fold gain in diameter and 9.4-fold in volume. Histograms of the size distributions of alginate- and chitosan-based U-4CR droplets and microgels are presented in the Appendix Figure A3.

### 3.2. In-Depth Analysis of Hyaluronic Acid-Based U-4CR Microgels

#### 3.2.1. Screening Crosslinker Concentration and Molecular Weight in a Parameter Matrix

To screen the potential influence of PEG-diamine crosslinker length and concentration on the physicochemical properties of U-4CR microgels, a 3-by-3 parameter matrix is generated. For all samples, the amount of hyaluronic acid sodium salt is kept constant at 12 mg, dissolved in deionized water, and acidified with 2 M HCl. The parameter screening matrix screens the amount of the crosslinker PEG-diamine (8 mg, 12 mg, and 16 mg) against different molecular weights of 2000 g mol^−1^ (PEG-diamine 2K), 1000 g mol^−1^ (PEG-diamine 1K), and 600 g mol^−1^ (PEG-diamine 0.6K) (Table 4). For all hydrogel precursor solutions (referred to as the dispersed phase, DP, in the microfluidic experiments) screened in the matrix, a threefold amount of formaldehyde is added with respect to the molar amount of PEG-diamine.

The precursor solutions are injected into the microfluidic flow cell and microdroplets, containing the hydrogel precursors, form at the first junction, whereas at the second junction *tert*-butyl isocyanide is added to diffuse into the microdroplets to induce the U-4CR crosslinking reaction. After 48 h of reaction time, the emulsion is broken by the addition of PFO (20% *v*/*v* in Novec 7500), which drives the transfer of microgels into a purely aqueous phase (deionized water). For each of the three molecular weights of crosslinkers (2K, 1K, 0.6K), we observe indications for trends of the swelling ratio depending on the amount of crosslinker used for microgel preparation. These trends differ depending on the crosslinker length (Table 5). In the case of PEG-diamine 2K, a decrease in the swelling ratio is observed. In contrast to this finding, the swelling ratio increases with decreasing mass of PEG-diamine 1K. A third behavior is shown by the U-4CR microgels with PEG-diamine 0.6K. Here, the swelling ratio stays constant while varying the crosslinker amount. The formula for the calculation of the error propagation of the size increase can be found in Appendix B.

These three distinct behaviors for each crosslinker can be understood and explained in more detail by determining the Young’s moduli of the microgels by colloidal probe atomic force microscopy (CP-AFM) (Section 3.2.2) and optical characterization methods (Section 3.2.3 and Section 3.2.4).

#### 3.2.2. Young’s Moduli of Hyaluronic Acid-Based U-4CR Microgels Determined by CP-AFM

To quantify the impact of crosslinker variations in terms of molecular weight and concentration on the mechanical properties of the microgels, the different U-4CR gels are studied by indentation by means of CP-AFM [47]. This method allows for determining the Young’s moduli *E* of the microgels with high accuracy. To achieve reliable results, the colloidal probe (native silica, *d* = 20 µm) is aligned directly over the apex of the microgel. This apex is located with a spatially resolved grid of force-distance curves resulting in a topographical image of the spherical gel. Subsequently, five approach-retract cycles are conducted to obtain force-deformation (F-D) curves for 20 microgels of each type. The Young’s modulus can be extracted from the slope of these F-D curves by several theories available for contact mechanics. The Johnson-Kendall-Roberts (JKR) theory was used to take the observed adhesion forces acting between the colloidal probe and the microgels into account, because the frequently used Hertz model neglects inter-particle forces.

**Figure 5:**
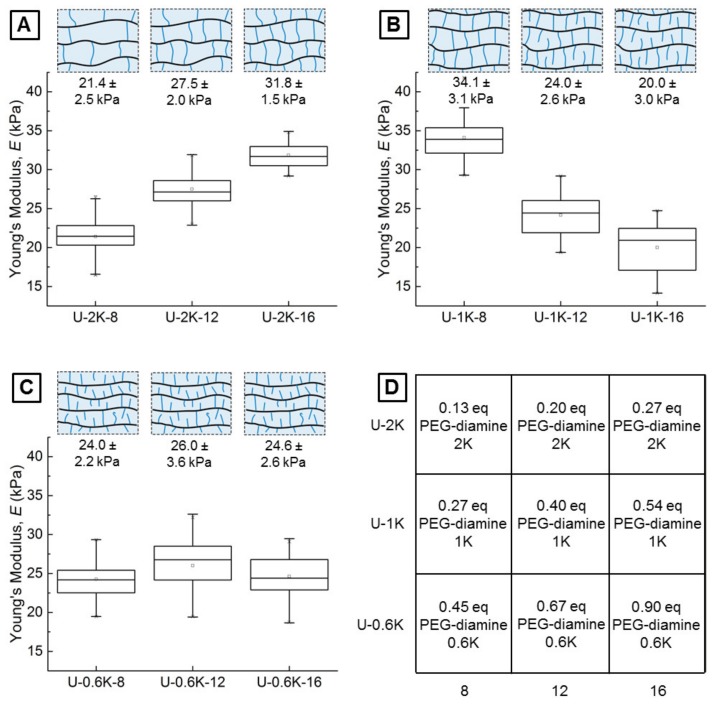
(**A**) Boxplots of the Young’s moduli of U-2K-type microgels obtained from evaluating five F-D curves of 20 microgels each, and schematic assumption of corresponding hydrogel network structures. (**B**) Analogous to (**A**) for U-1K-type microgels. (**C**) Analogous to (**A**) for U-0.6K-type microgels. (**D**) Matrix of microgel variations with corresponding equivalents of homobifunctional PEG-diamine crosslinker added to the precursor solution.

The Young’s moduli of the U-2K-type microgels show the expected behavior with an increase from 21.4 ± 2.5 kPa over 27.5 ± 2.0 kPa to 31.8 ± 1.5 kPa when increasing the crosslinker amount from 8 mg to 12 mg or 16 mg, respectively, which corresponds to a crosslinker concentration in the initial pre-gel reaction mixtures of 20 mmol L^−1^, 30 mmol L^−1^ and 40 mmol L^−1^. This finding can be explained by the increasing number of crosslinking sites, formed during the U-4CR crosslinking reaction, which are responsible for the networks stiffness. It is assumed that a high percentage of homobifunctional crosslinker is connected at both terminal ends of the PEG-diamine chain to the polysaccharide backbone, creating the hydrogel network. Since the same mass of crosslinker is used for the different types of microgels, the relative equivalents of crosslinker to hyaluronic acid are increased by a factor of two when the crosslinker molecular weight is halved from 2K to 1K (cf. Figure 5D). In this case, the Young’s moduli decrease with increasing crosslinker amounts from 34.1 ± 3.1 kPa over 24.0 ± 2.6 kPa to 20.0 ± 3.0 kPa. As it can be seen in Figure 5D, U-2K-16- and U-1K-8-type microgels sharing the same amount of crosslinker equivalents (0.27) result in comparable Young’s moduli. 0.27 equivalents of 1K PEG-diamine give a slightly higher modulus, which can be explained by formation of a denser hydrogel network and therefore a lower elasticity because of shorter crosslinking sites. Comparing U-2K- and U-1K-type HA microgels formed by Ugi-4CR lead to the assumption that above 0.27 equivalents of homobifunctional crosslinker, a transition from double-sided to single-sided connection of crosslinkers occurs due to improved availability of functional groups at the crosslinker ends. Another reason for this behavior could be an increasing probability of intramolecular crosslinking at high crosslinking densities. This assumption is supported by the findings of Jeon et al., who have investigated the mechanical properties of hyaluronic acid-based bulk gels, crosslinked by varying the amount of a PEG-diamine incorporated into the network via carbodiimide chemistry [48]. The authors observe exactly the same behavior of increasing Young’s moduli *E* with increasing crosslinker amount until reaching an inflection point, above which a further increase leads to a decrease in *E*. The network schemes depicted in Figure 5 serves to illustrate these characteristics of gel formation.

The *E* moduli of the U-0.6K-type microgels reveal a third behavior. Here, increasing the crosslinker amount does not lead to a variation of the *E* modulus (Figure 5C). When theoretically targeting 90% of the carboxylic acid groups of the hyaluronic acid in case of U-0.6K-8, a plateau is reached, from which a further increase of crosslinker molecules no longer leads to a change of network properties for reasons discussed above. The modest increase of the *E* moduli of U-0.6K-type microgels in contrast to the U-1K-16-type microgels is, as already discussed, attributed to an increasing network density caused by shorter crosslinks of the PEG-diamine.

Indications for a trend observed in the Young’s modulus E for different crosslinker lengths is also observed for the size increase of droplet-to-microgel transition. This correlation suggests that these two parameters are closely connected. The connection of E modulus and size increase due to swelling was previously reported by Yazici et al. [49].

#### 3.2.3. Analysis of Hyaluronic Acid-Based U-4CR Microgels by Optical Techniques

**Figure 6:**
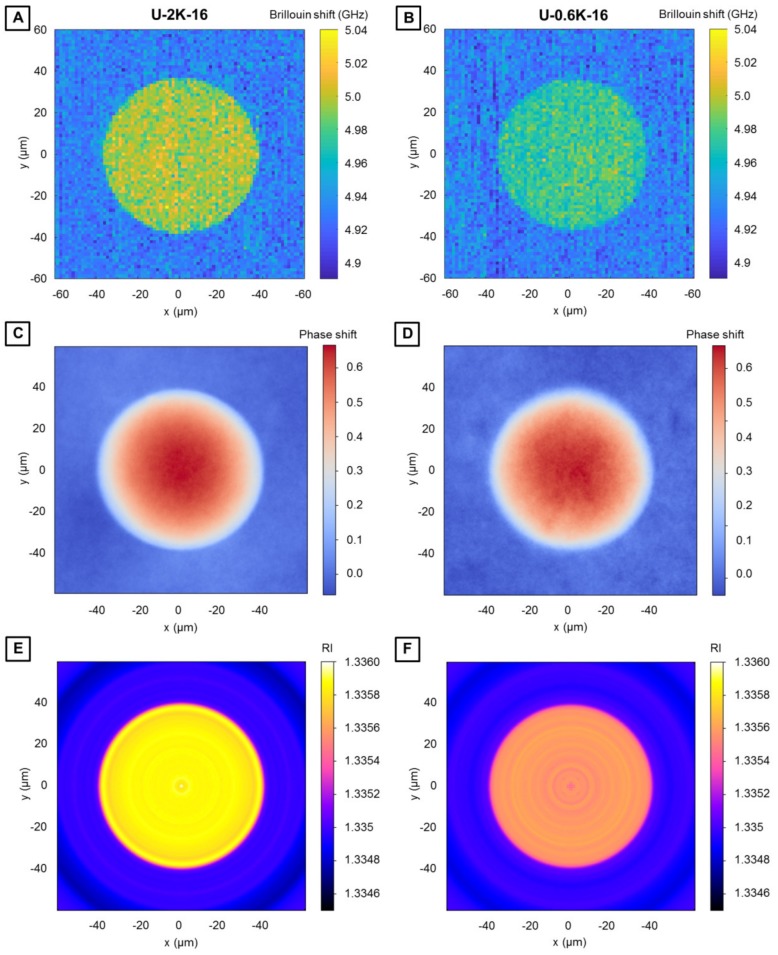
(**A**,**B**) Brillouin shift (*ν_B_*) image of a single microgel of U-2K-16 type (**A**) and U-0.6K-16 type (**B**). (**C**,**D**) Quantitative phase image of a single microgel for refractive index (RI) determination of U-2K-16 type (**A**) and U-0.6K-16 type (**B**). Color scale bar: Phase shift in radians. (**E**,**F**) Cross-section of a tomographic RI reconstruction from the quantitative phase images (**C**) and (**D**) (by assuming spherical symmetry) of U-2K-16 type (**E**) and U-0.6K-16 type (**F**).

**Figure A4:**
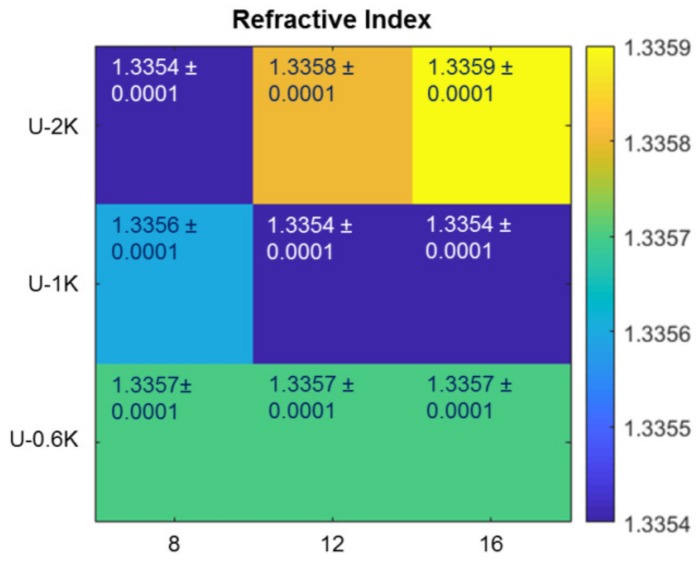
Heat map of measured refractive indices of crosslinker variation matrix types of hyaluronic acid-based U-4CR microgels (U = Ugi; 0.6K, 1K, 2K = x 1000 g mol^−1^, molecular weight of cross-linker; 8, 12, 16 = x mg cross-linker).

Optical analysis techniques, more precisely confocal Brillouin microscopy and quantitative phase imaging, are carried out to obtain detailed insights into the microgel network properties. Since the microgel fabrication method contains a step of diffusion of the isocyanide, as the fourth component of the U-4CR, through the surfactant layer into the aqueous droplet, questions arise, if a homogeneously crosslinked microgel, a microgel with a stiffness gradient from the outside to the core, or some kind of microcapsule forms. Brillouin microscopy is applied to spatially resolve the mechanical properties within the U-4CR microgels for a detailed characterization in terms of homogeneity, similar to a recent study demonstrating this for microgels consisting of poly(acrylamide) [50]. This technique is based on the inelastic scattering process between incident photons and acoustic phonons within the sample. The Brillouin shift is measured, which is related to the longitudinal modulus, the refractive index, and the density of the sample. By automatically translating the microscope stage, exemplarily spatially resolved maps of two representative hyaluronic acid-based microgel types, made from PEG crosslinkers with a molecular weight of 2K and 0.6K, are acquired (Figure 6A,B). The refractive indices of the hyaluronic acid-based U-4CR microgels are determined with a quantitative phase-imaging camera according to Schürmann et al. [39]. The corresponding heat map of refractive indices of U-4CR microgels is displayed in the Appendix Figure A4. Determined refractive indices of all investigated U-4CR microgels have values of 1.335× ± 0.0001, corresponding essentially to the refractive index of water (refractive index of 1.333), which is expected from the high water content of the gels of around 98%. The Brillouin shift maps (Figure 6A,B), quantitative phase images (Figure 6C,D), and tomographic refractive index reconstructions from the quantitative phase images (Figure 6E,F) of U-2K-16 and U-0.6K-16, both microgels with 16 mg of PEG-diamine crosslinker, prove homogeneity of the network structure all over the investigated microgel for both types. Consequently, the droplet microfluidic technique is well suited for the fabrication of homogeneous U-4CR microgels without gradients in terms of density or mechanics. A direct comparison of the Brillouin shift maps in Figure 6A,B and refractive index reconstructions in Figure 6E,F reveals the higher overall Brillouin shift and refractive index of U-2K-16 compared to U-0.6K-16. This behavior was already observed for the Young’s modulus by CP-AFM.

**Figure A5:**
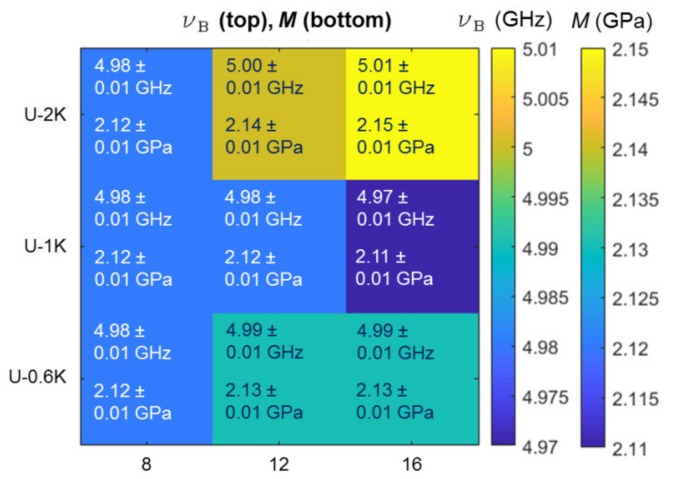
Heat map of measured Brillouin shifts (*ν_B_*) and computed longitudinal moduli *M* of crosslinker variation matrix types of hyaluronic acid-based U-4CR microgels.

**Figure A6:**
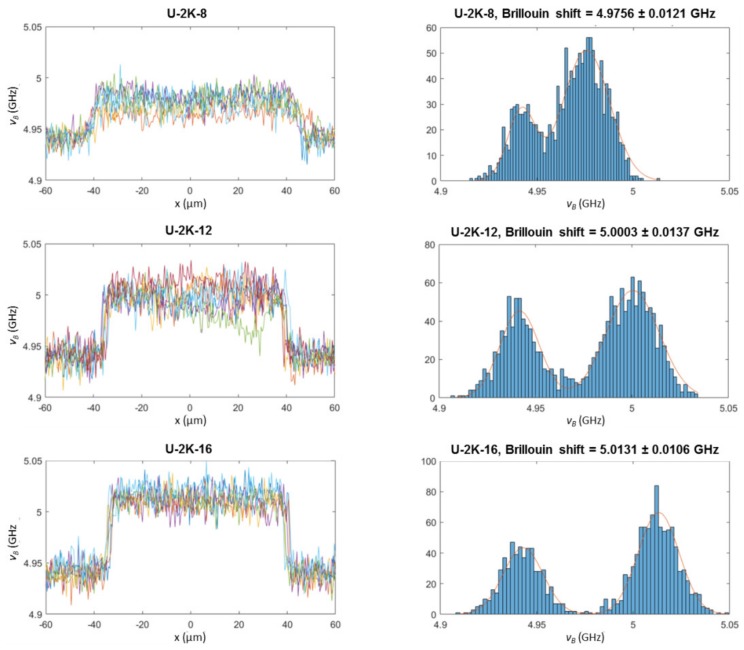
Brillouin shift line scans of U-2K microgels (**left**) and corresponding histograms (**right**).

**Figure A7:**
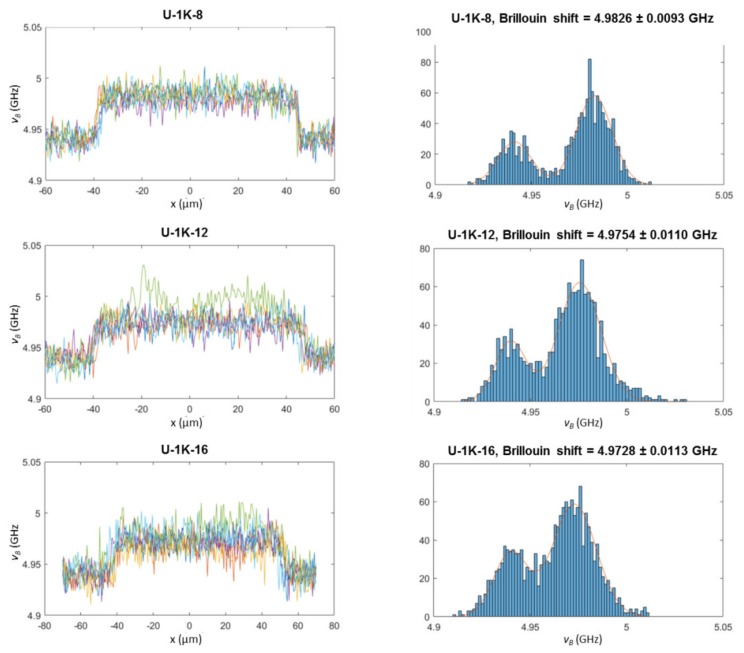
Brillouin shift line scans of U-1K microgels (**left**) and corresponding histograms (**right**).

**Figure A8:**
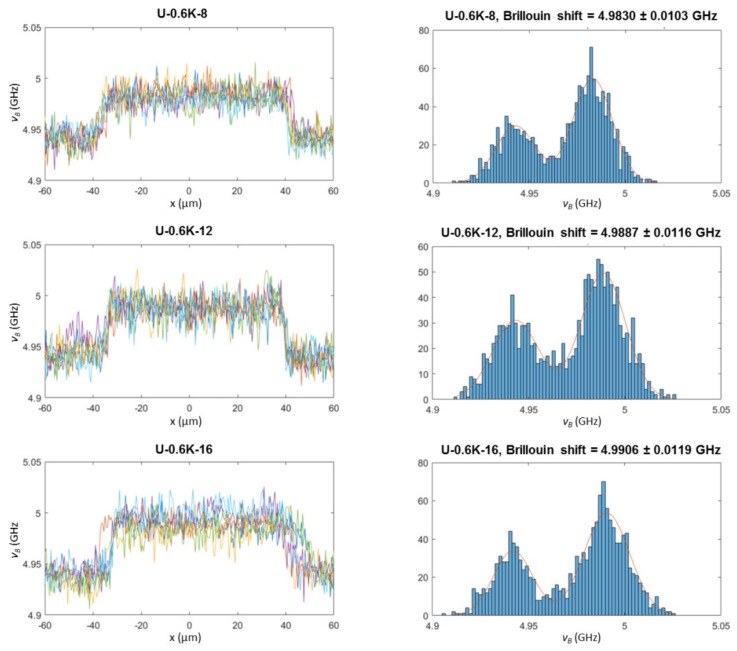
Brillouin shift line scans of U-0.6K microgels (**left**) and corresponding histograms (**right**).

To investigate the results of the effects of the crosslinker on microgel mechanics, line scans across the center of U-4CR microgels are performed. The Brillouin shifts determined from these scans are averaged and displayed in the Appendix Figure A5. Raw data and corresponding histograms of these line scans can be found for all hyaluronic acid-based U-4CR microgels in the Appendix Figure A6, Figure A7 and Figure A8. The absolute density of the microgels is calculated from the refractive indices [36]. Since the mechanical properties are described by the longitudinal modulus, the refractive index, and the mass density, the longitudinal modulus is computed based on the previously determined values according to Schlüßler et al. [36] (Appendix Figure A5).

#### 3.2.4. Diffusion Coefficients Determined by FCS and Conclusions on Microgel Network Structure

**Figure A9:**
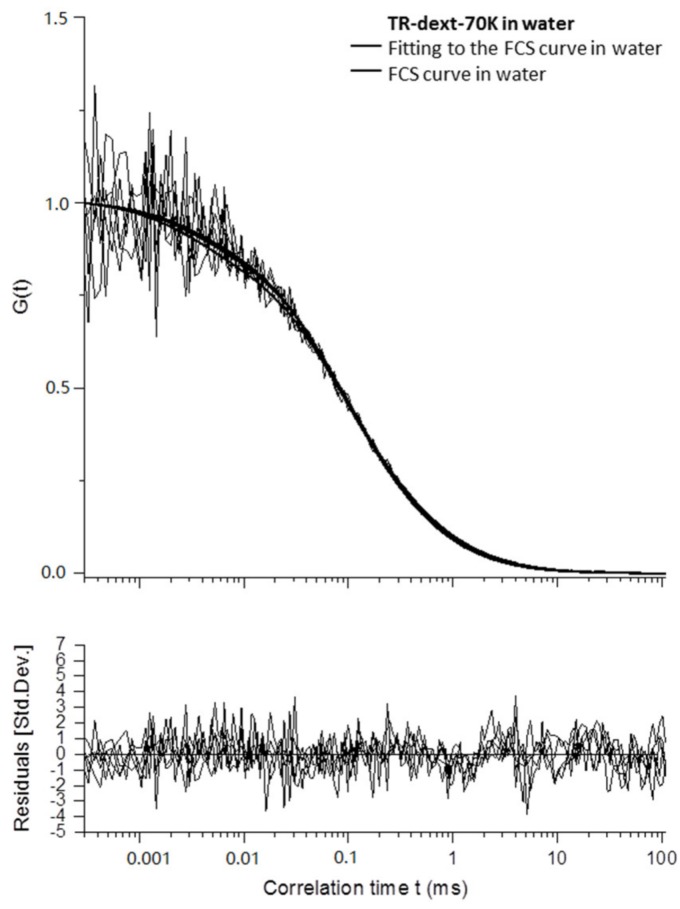
FCS curves and corresponding fits based on the molecular probe tetramethylrhodamine-dextran (70 kDa) in water. The lower panel shows the residuals of the fits.

**Figure A10:**
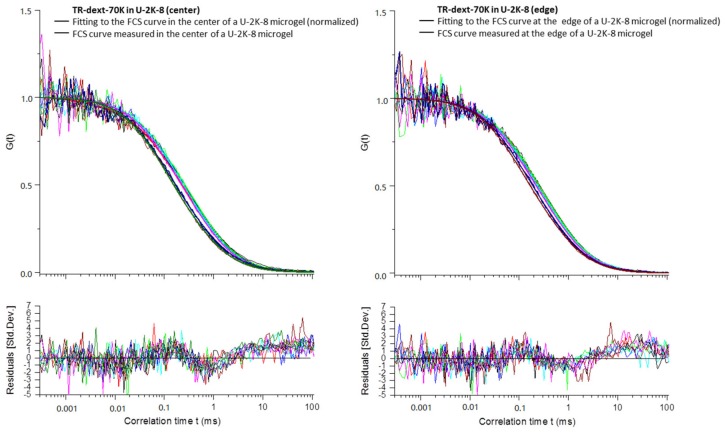
FCS curves and corresponding fits of tetramethylrhodamine-dextran (70 kDa) measured in the center (left) and at a position 25 µm away from the center position (referred to as “edge” in the plots) of U-2K-8 microgels.

**Figure A11:**
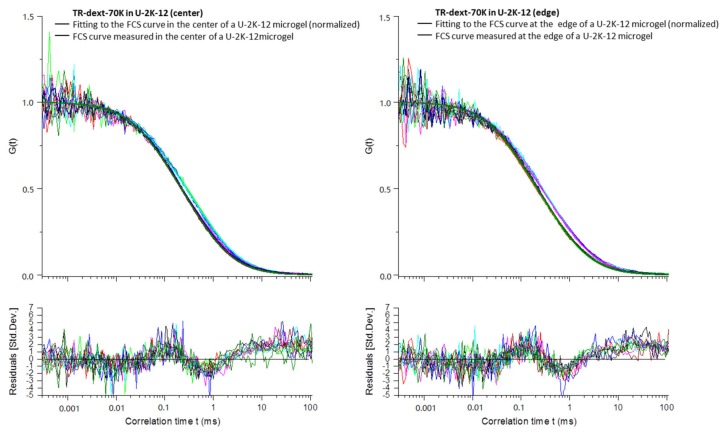
FCS curves and corresponding fits of tetramethylrhodamine-dextran (70 kDa) measured in the center (left) and at a position 25 µm away from the center position (referred to as “edge” in the plots) of U-2K-12 microgels.

**Figure A12:**
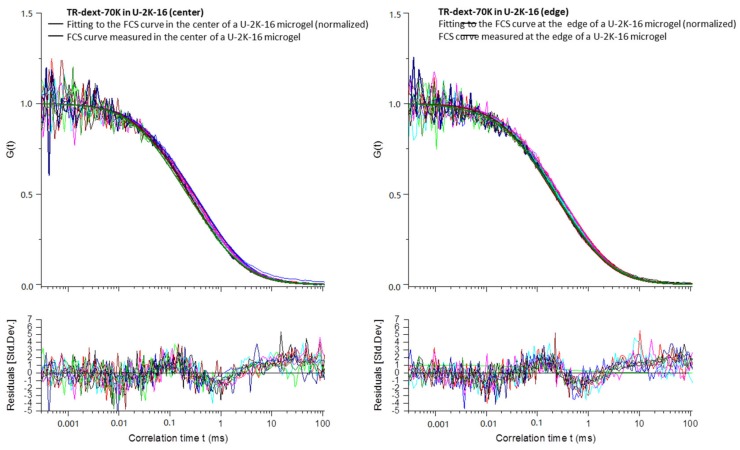
FCS curves and corresponding fits of tetramethylrhodamine-dextran (70 kDa) measured in the center (left) and at a position 25 µm away from the center position (referred to as “edge” in the plots) of U-2K-16 microgels.

For a more detailed investigation of the crosslinker influence on microgel network formation and thus on network porosity, fluorescence correlation spectroscopy is exemplarily carried out on U-2K-type hyaluronic acid-based microgels made by Ugi-4CR. They confirm the structural homogeneity already shown with the confocal Brillouin microscopy. FCS measurements are conducted at a height of approximately 40 µm in the center of the microgel and at a lateral distance of approximately 25 µm from the center. The autocorrelation curves do not significantly differ from each other, thus, underlining the structural homogeneity. At least 18 different measurements are carried out for each microgel type (8, 12, and 16 mg PEGdiamine crosslinker), and additionally, compared to the diffusivity of the fluorescent FCS targets in an area outside of the microgels as reference. Results of the FCS measurements are shown in Table 6. The FCS curves and the corresponding fits of the autocorrelation functions of tetramethylrhodamine-dextran (70 kDa) in water (Appendix Figure A9) and in U-2K-type microgels can be found in the Appendix (Appendix Figure A10, Figure A11 and Figure A12).

FCS measurements detect not only the diffusion of the fluorescent probe tetramethylrhodamine-dextran, but also free fluorophores. Therefore, each FCS curve had to be fitted with two diffusion components. Additionally, to account for the photophysics of the fluorophore, triplet kinetics was taken into account (see Materials and Methods for details). Thus, diffusion time *t*_1_ and diffusion coefficient *D*_1_ are determined for the tetramethylrhodamine labelled dextranes, and parameters *t*_2_ and *D*_2_ for the free tetramethylrhodamine. In order to reduce the number of parameters to be fitted, it is assumed that the tetramethylrhodamine free molecules (non-labelling dextranes) do not change their diffusion times inside the microgels with respect to their values in free solution. Therefore, the ratios of diffusion coefficients *D*_2_/*D*_2_^sol^ of free dye in the microgel and in solution are set to 1. Under these conditions, the fitting of the FCS curves show that for the diffusion of dextran-bound fluorophores an impact due to the microgel network properties is detected. The diffusion coefficients *D*_1_ decrease with increasing amount of crosslinker in the initial pre-gel reaction mixture from 45.0 ± 3.0 µm^2^ s^−1^ for U-2K-8 over 41.6 ± 4.3 µm^2^ s^−1^ for U-2K-12 to 40.1 ± 2.6 µm^2^ s^−1^ for U-2K-16. This trend can be explained by the creation of a denser network with higher crosslinker concentration, resulting in more polymer chains per unit of volume and thereby smaller pore sizes affecting the diffusion of larger probe molecules. The diffusion coefficient ratio *D*_1_/*D*_1_^sol^ reveals that there is a 15%, 22% and 24% decrease for U-2K-8, U-2K-12, and U-2K-16, respectively, of the diffusion coefficient inside the microgel compared to the values in solution.

**Figure 7:**
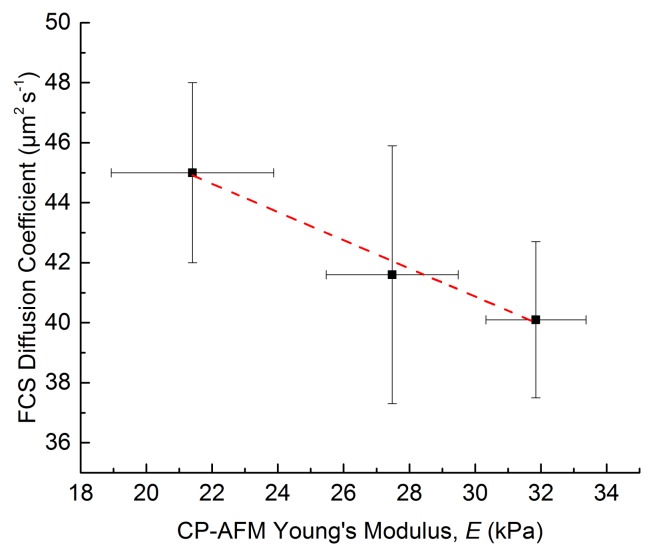
Plot of CP-AFM Young’s moduli *E* versus diffusion coefficients as determined by FCS, linearly fitted for U-2K-type microgels based on hyaluronic acid.

The FCS results show good correlation with the data of the CP-AFM measurements. Plotting the CP-AFM Young’s moduli E versus the diffusion coefficients as determined by FCS (Figure 7) yields a linear relationship with a coefficient of determination R^2^ > 0.98. Thus, these findings provide a hint that a higher Young’s modulus is accompanied by lower diffusion coefficients, supporting the presumption that a higher network density reduces the diffusion coefficient of molecules inside the hydrogel network.

### 3.3. Introduction of Functionality in Chitosan-Based U-4CR Microgels

#### 3.3.1. Biotin-Functionalized, Chitosan-Based U-4CR Microgels

The versatility of multicomponent reactions for microgel synthesis is demonstrated by fabricating biotin-functionalized, chitosan-based microgels via U-4CR. Here, chitosan is crosslinked by PEG-dialdehyde and functionalized in situ by incorporating heterobifunctional carboxylic acid-PEG-biotin into the hydrogel network instead of acetic acid in conventional microgel formation (cf. Section 2.3.6).

**Figure 8:**
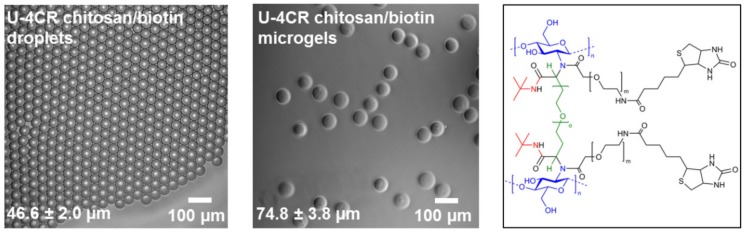
Phase-contrast microscopy images of emulsion droplets (**left**) and corresponding chitosan-based microgels formed by U-4CR utilizing carboxylic acid-PEG-biotin as functional building block including mean and standard deviation (**middle**), chemical structure of functional chitosan-based microgels focusing on the crosslinking site presenting biotin moieties (**right**).

**Figure A13:**
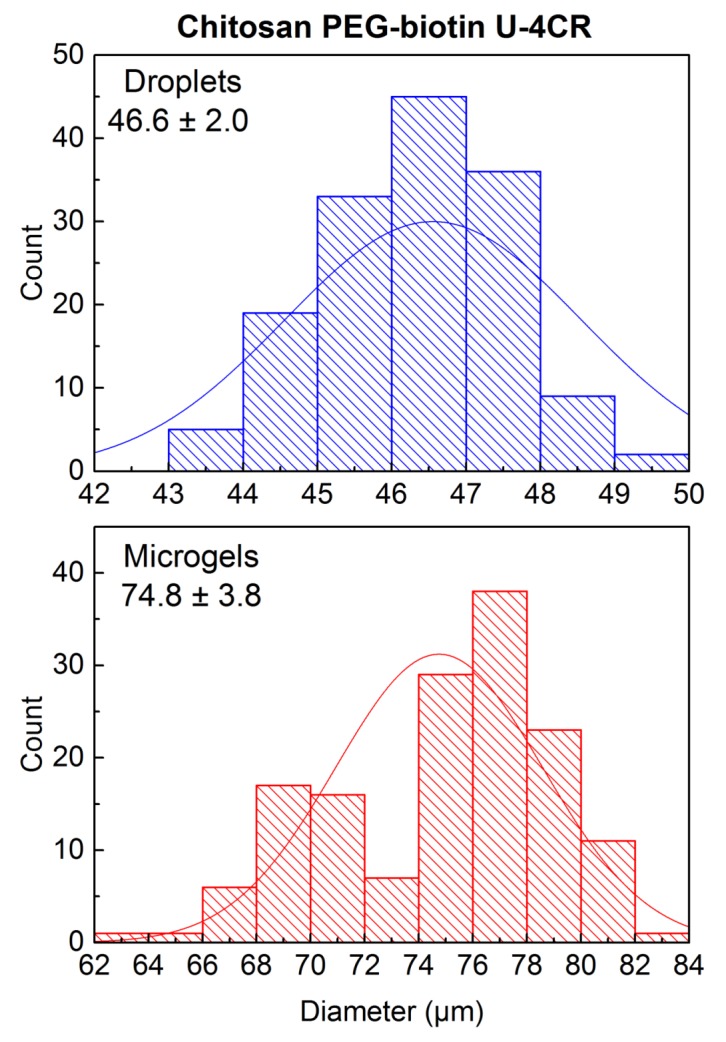
Size distribution histograms with additional mean and standard deviation for droplets and microgels of chitosan-based, biotin-functionalized U-4CR microgels.

Based on the protocol for non-functionalized chitosan-based microgels, the carboxylic component acetic acid was substituted by equimolar amounts of carboxylic acid-PEG-biotin. 48 h after droplet-assisted production of the microdroplets by the microfluidic setup shown in Figure 3, the collected emulsion is broken by addition of PFO (20% *v*/*v* in Novec 7500) to transfer the microgels into deionized water. Droplets of 46.6 ± 2.0 µm translate into microgels, 74.8 ± 3.8 µm in diameter (see Figure 8), corresponding to a 1.6-fold gain in diameter and a 6.4-fold increase in volume, which is less compared to 2.4–fold and 9.4-fold of previously produced non-functionalized chitosan gels. This finding suggests that the coupling efficiency of the U-4CR is higher with carboxylic acid-PEG-biotin, compared to the reaction with acetic acid, which leads to a higher degree of crosslinking and therefore to a lower swelling ability in water. Size distribution histograms of droplets and microgels can be found in the Appendix Figure A13.

#### 3.3.2. Proof of Biotin Availability in Chitosan-Based Microgels by Streptavidin Conjugation

**Figure 9:**
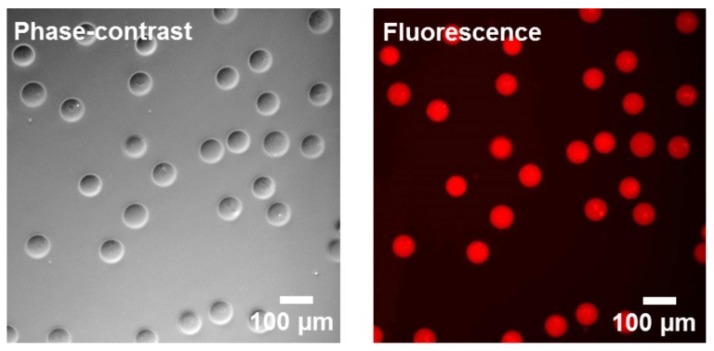
Phase-contrast image and fluorescence microscopy image (TXR channel) of chitosan-based, biotin-functionalized microgels made by U-4CR with attached Alexa Fluor 555-streptavidin.

To prove the availability of incorporated biotin moieties inside the chitosan-based microgels, fluorescent Alexa Fluor 555-streptavidin is added to the microgel suspension. Biotin-streptavidin affinity is highly specific and known as one of the strongest non-covalent biological interactions [52]. After shaking for 30 min at room temperature, the streptavidin conjugate is quantitatively bound to the microgels, as proven by fluorescence microscopy of the microgel dispersion (Figure 9) and its supernatant.

#### 3.3.3. Application of Biotin-Functionalized, Chitosan-Based U-4CR Microgels for Enzyme Immobilization

**Figure 10:**
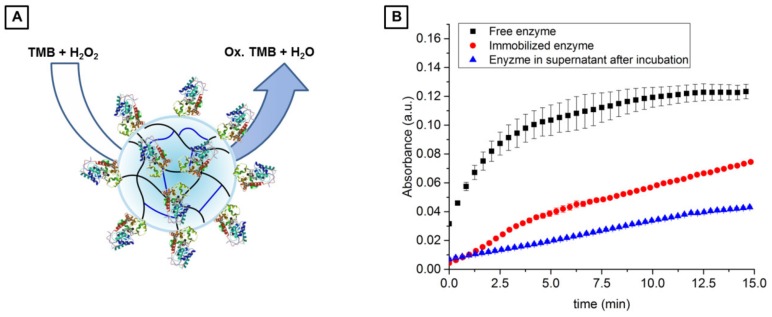
(**A**) Scheme of HRP immobilized via streptavidin-biotin interaction on the chitosan-based U-4CR microgel network, and enzymatic conversion of colorless TMB forming blue-colored, oxidized TMB, a cationic radical. (**B**) Absorbance measurements investigating the formation of blue-colored TMB by free HRP in solution (black), HRP immobilized on chitosan-based U-4CR microgels (red), and free HRP remaining in solution after the incubation time of the immobilization reaction (blue). In case of the experiments of free and immobilized HRP the initial concentration of the enzyme was 0.05 µM.

To prove that functionalized U-4CR microgels are well suited for application in the field of cell-free biotechnology, the immobilization of a streptavidin-tagged enzyme was exemplarily investigated. Since horseradish peroxidase (HRP) is an enzyme widely used in biochemistry applications [53], it was used in this work as a model enzyme. It catalyzes the conversion of chromogenic substrates, for instance 3,3′,5,5′-tetramethylbenzidine (TMB), into colored products allowing for straight-forward detection of reaction progress. A microgel suspension of biotin-functionalized, chitosan-based U-4CR microgels is incubated with a conjugate of HRP and streptavidin overnight at 4 °C. Unreacted enzyme is then removed by repeated washing with water to ensure that only enzyme, which is coupled via streptavidin-biotin interaction, remains in and on the microgels. To evaluate coupling efficiency, enzymatic activity of the immobilized HRP is compared to free enzyme in solution at the same concentration as added to the microgels for functionalization. For that, the conversion of TMB and hydrogen peroxide yielding oxidized TMB in form of a blue-colored cationic radical derivative is analyzed (depicted in Figure 10A) by light absorption of this derivate at a wavelength of 643 nm. The results of this activity assay are shown in Figure 10B, which reveal that microgel-coupled HRP is still active after immobilization, resulting in 60% substrate conversion compared to free enzyme after a reaction time of 15 min (Figure 10B). The supernatant removed from the enzyme-functionalized microgels exhibits a substrate conversion of 35% compared to reference experiment with free enzyme. This finding indicates that the enzyme retains its activity nearly quantitatively after immobilization. The remaining slight decrease in activity could be a result of conformational changes due to immobilization of the enzyme in a polymer matrix, however, immobilization occurs via streptavidin and thus the active center of HRP should not be affected. Therefore, it is more likely that the small decrease in activity arises from a loss of active enzyme or limited access to the enzymes active site due to attachment to the wall of the reaction vessel.

## 4. Conclusions

Droplet microfluidics-assisted synthesis of polysaccharide microgels via Passerini three-component and Ugi four-component reactions has been introduced. In both reactions, diffusion of the isocyanide component from the continuous phase through the surfactant layer into the microdroplets initiates the microdroplet-to-microgel conversion. Especially, the U-4CR is well suited for the fabrication of diverse polysaccharide microgels based on hyaluronic acid, alginate or chitosan with a narrow size distribution for each material basis. Exemplary in-depth analysis of hyaluronic acid-based gels by CP-AFM, Brillouin microscopy, quantitative phase imaging, and FCS reveals high network homogeneity of investigated samples. Furthermore, the impact of crosslinker concentration has been examined by these methods, indicating that higher Young’s moduli are paired with smaller pore sizes and therefore lower diffusion coefficients.

Moreover, we demonstrate the ability of the U-4CR to fabricate functional microgels in a modular fashion using the example of biotin-functionalized, chitosan-based microgel preparation. Here, the availability of biotin moieties attached to the hydrogel network is confirmed by attachment of fluorescent streptavidin. Additionally, biotin-streptavidin interactions can be utilized for immobilization of streptavidin-conjugated enzymes, exemplified by HRP, which retains its activity after immobilization. Based on the modular principle of the P-3CR and U-4CR, exchange of individual reaction components enables simultaneous crosslinking and (multi-)functionalization towards the comparatively simple synthesis of tailor-made microgels for highly specific applications. 

## Figures and Tables

**Table 1:** Amounts of PEG-diamine 2000 g mol^−1^ and formaldehyde 37% for microfluidic experiments ^a^.

Sample	PEG-Diamine (M_W_: 2000 g mol^−1^)	Formaldehyde 37%
U-2K-8	8 mg; 0.13 eq	0.9 µL; 0.4 eq
U-2K-12	12 mg; 0.20 eq	1.4 µL; 0.6 eq
U-2K-16	16 mg; 0.27 eq	1.8 µL; 0.81 eq

^a^ Equivalents (eq) related to molarity of hyaluronic acid.

**Table 2:** Amounts of PEG-diamine 1000 g mol^−1^ and formaldehyde 37% for microfluidic experiments ^a^.

Sample	PEG-Diamine (M_W_: 1000 g mol^−1^)	Formaldehyde 37%
U-1K-8	8 mg; 0.27 eq	1.8 µL; 0.81 eq
U-1K-12	12 mg; 0.40 eq	2.7 µL; 1.21 eq
U-1K-16	16 mg; 0.54 eq	3.6 µL; 1.61 eq

^a^ Equivalents (eq) related to molarity of hyaluronic acid.

**Table 3:** Amounts of PEG-diamine 600 g mol^−1^ and formaldehyde 37% for microfluidic experiments ^a^.

Sample	PEG-Diamine (M_W_: 600 g mol^−1^)	Formaldehyde 37%
U-600-8	8 mg; 0.45 eq	3.0 µL; 1,34 eq
U-600-12	12 mg; 0.67 eq	4.5 µL; 2.02 eq
U-600-16	16 mg; 0.90 eq	6.0 µL; 2.69 eq

^a^ Equivalents (eq) related to molarity of hyaluronic acid.

**Table 4:** Samples in the crosslinker variation matrix for screening the crosslinker amount and length.

Crosslinker Length	8 mg	Crosslinker Amount 12 mg	16 mg
**2000 g mol^−1^**	U-2K-8	U-2K-12	U-2K-16
**1000 g mol^−1^**	U-1K-8	U-1K-12	U-1K-16
**600 g mol^−1^**	U-0.6K-8	U-0.6K-12	U-0.6K-16

**Table 5:** Droplet and microgel diameter, and corresponding size increases of hyaluronic acid-based U-4CR microgels.

Sample	Droplet Diameter	Microgel Diameter	Size Increase ^a^
**U-2K-8**	44.5 ± 1.2 µm	89.2 ± 4.2 µm	2.0 ± 0.1
**U-2K-12**	42.6 ± 1.2 µm	72.9 ± 2.1 µm	1.7 ± 0.1
**U-2K-16**	40.6 ± 1.3 µm	74.7 ± 1.8 µm	1.8 ± 0.1
**U-1K-8**	44.7 ± 2.7 µm	79.7 ± 2.7 µm	1.8 ± 0.1
**U-1K-12**	44.9 ± 1.1 µm	83.9 ± 2.1 µm	1.9 ± 0.1
**U-1K-16**	46.3 ± 1.1 µm	90.8 ± 1.9 µm	2.0 ± 0.1
**U-0.6K-8**	47.5 ± 1.9 µm	78.7 ± 4.1 µm	1.7 ± 0.1
**U-0.6K-12**	45.7 ± 1.0 µm	75.7 ± 2.5 µm	1.7 ± 0.1
**U-0.6K-16**	44.0 ± 1.0 µm	75.2 ± 4.1 µm	1.7 ± 0.1

^a^*D*_Microgels_/*D*_Droplets_.

**Table 6:** Diffusion coefficients and diffusion times of tetramethylrhodamine-dextran (70 kDa) in U-2K-type microgels.

Exp.^a^	Type	*D*_1_ (µm^2^ s^−1^)	*D*_2_ (µm^2^ s^−1^)	*D*_1_/*D*_1_^sol^	*D*_2_/*D*_2_^sol^	*t*_1_ (ms)	*t*_2_ (ms)
Mean	U-2K-8	45.0 ± 3.0	363 ^c^	0.85	1.00	0.66 ± 0.04	0.082 ^c^
Mean	U-2K-12	41.6 ± 4.3	363 ^c^	0.78	1.00	0.73 ± 0.08	0.082 ^c^
Mean	U-2K-16	40.1 ± 2.6	363 ^c^	0.76	1.00	0.75 ± 0.05	0.082 ^c^
Mean	solution	53.0 ± 12.0	366 ± 24	-	-	0.58 ± 0.16	0.082 ^c^
Lit.^b^	solution	37.0 ± 6.6	412 ± 18	-	-	-	-

^a^ All experiments data corresponding to 22 ± 1.0 °C. ^b^ Data corresponding to 23 ± 1.5 °C published by Zhang et al. [51]. ^c^ Parameter fixed.

## Appendix A

**Note:** P-3CR (= Passerini three-component reaction), U-4CR (= Ugi four-component reaction)

## Appendix B

### Calculation of Error Propagation of Size Increase during Microdroplet-to-Microgel Transition

The size increase *I*_S_ of conversion of microdroplets to microgels is calculated by the quotient of mean microgel diameter *D*_Microgels_ to mean droplet diameter *D*_Droplets:_

(1) $$I_{s}=\frac{D_{\mathrm{Microgels}}}{D_{\mathrm{Droplets}}}$$

(2) $$\sigma_{I_{S}}=\sqrt{{\left(\frac{1}{D_{\mathrm{Droplets}}}\sigma_{\mathrm{Microgels}}\right)}^{2}+{\left(-\frac{D_{\mathrm{Microgels}}}{D_{\mathrm{Droplets}}{}^{2}}\sigma_{\mathrm{Droplets}}\right)}^{2}}$$
